# Application of the natural orifice specimen extraction surgery I-type E method combined with 3D laparoscopy in sphincter-preserving surgery of low rectal cancer

**DOI:** 10.3389/fsurg.2022.972258

**Published:** 2022-09-07

**Authors:** Liu Maoxi, Guo Xingyu, Bai Wenqi, Jiang Bo

**Affiliations:** Department of Colorectal Surgery, Shanxi Provincial Cancer Hospital, Taiyuan, China

**Keywords:** NOSES I-type E method, 3D laparoscopy, low rectal cancer, sphincter-preserving, surgery

## Abstract

**Purpose:**

Analysis of the clinical efficacy of the application of the NOSES I-type E method combined with 3D laparoscopy in sphincter-preserving surgery of low rectal cancer.

**Method:**

A retrospective analysis of 109 patients who underwent laparoscopic low rectal cancer surgery for anus preservation without preventive stoma admitted to the Department of Colorectal Surgery in Shanxi Provincial Cancer Hospital between January 2017 and May 2019. The 109 cases comprised 52 cases treated with the NOSES I-type E method (NOSES I-type E group) and 57 cases treated with the Dixon method (Dixon group). In the NOSES I-type E group, 25cases underwent 3D laparoscopic surgery (group A) and 27 cases underwent 2D laparoscopic surgery (group B). The general clinical data, perioperative indicators, three-day postoperative pain score, postoperative pathological conditions, complications, return visit to assess the 1-year postoperative anal function, 3-year local recurrence and distant metastasis, and survival were compared among the groups.

**Result:**

The distance between the tumor and the anal verge was significantly different between NOSES I-type E group and the Dixon group (*P *<* *0.05), while there was no significant difference between group A and group B (*P *> 0.05). The exhaust time, eating time, drainage tube removal time, hospitalization costs, hospitalization time, and the number of days of analgesic administration were significantly different between NOSES I-type E group and the Dixon group (*P *< 0.05), while group A had no significant difference compared to group B (*P *> 0.05). There were significant differences in difficulty urinating between group A and B (*P *< 0.05), while there was no significant difference between NOSES I-type E group and the Dixon group (*P* > 0.05). Anastomotic leakage in NOSES I-type E group were significantly lower than those in the Dixon group (*P *< 0.05), while there was no significant difference between group A compared to group B (*P *> 0.05). Anal stenosis, rectal Prolapse and colon retraction in NOSES I-type E group were significantly higher than those in Dixon group (*P *<* *0.05), there was no significant difference between group A compared to group B (*P *> 0.05). Anastomotic bleeding in Dixon group occurred in higher frequency than in NOSES I-type E group (*P *<* *0.05). The pain scores of patients in NOSES I-type E group in the first three days after operation were significantly lower than those in Dixon group (*P *< 0.05),while there was no significant difference between group A and group B (*P *> 0.05). There were no significant differences in postoperative pathology, 1-year postoperative anal function score, 3-year recurrence rate and overall survival rate among the groups (*P *>* *0.05).

**Conclusion:**

The NOSES I-type E method is a safe and effective sphincter-preserving operation for low rectal cancer and its combination with 3D laparoscopy may have better neurological protection which is worth of clinical application.

## Introduction

Rectal cancer is a common malignant tumor type of the digestive system and its morbidity and mortality is increasing. Low rectal cancer (less than or equal to 7 cm from the anal verge) accounts for about 60%–75% of all rectal cancers (1). It has always been a difficult and hot topic in clinical work to pay attention to preserving the anus and its function while pursuing a good survival rate for those patients. The technologies that have added great value to achieve this goal are laparoscopic and minimally invasive surgery technology (2). In recent years, 3D laparoscopy has been widely used in rectal cancer surgery. It combines the advantages of a clearer anatomy, a more precise neuroprotection and a strong depth with that of a three-dimensional sense and meanwhile has been accepted by the majority of colorectal surgeons as a useful tool. Therefore, its application in colorectal surgery has become increasingly popular.

For low rectal cancer anus preserving surgery, laparoscopy-assisted anterior rectal resection (Dixon) has achieved good results. Specimen collection through a natural orifice combines natural orifice endoscopic surgery and laparoscopic surgery. In recent years, the application of NOSES surgery has become increasingly accepted by colorectal surgeons (3). NOSES I techniques are divided into A, B, C, D, and E methods, of which the E subtype is the main surgical method for low rectal cancer in NOSES surgery (4). Nevertheless, the NOSES I-type E method, which combines laparoscopy with the modified Bacon method, as a sphincter-preserving surgical method for low rectal cancer, its indications, complications, anal function, and prognosis is still controversially discussed (5). However, major points of the NOSES surgery debate still seem to be total tumor resection and sterility (6). Moreover, its procedure is complex and difficult and thus, still needs to be continuously improved (7). Therefore, its comparison with the laparoscopic-assisted combined anterior rectal resection with sphincter-preserving surgery for low rectal cancer is one of the foci of clinical work.

As one of the largest colorectal cancer diagnosis and treatment center in China, the center has implemented the NOSES I type-E method to preserve the anus in patients with low rectal cancer since 2016 and has accumulated a considerable amount of data. However, there were rare reports on the application value of the NOSES I-type E method combined with 3D laparoscopy in sphincter-preserving surgery for low rectal cancer. To explore this issue, this study retrospectively analyzed a total of 109 patients who underwent low rectal cancer surgery using the NOSES I-type E method or the Dixon method in Shanxi Provincial Cancer Hospital in the time between January 2017 and May 2019 with anus-preserving surgery were divided into NOSES I-type E group and Dixon group according to the operation method, NOSES I-type E group further divided into 3D laparoscopy (group A) and 2D laparoscopy (group B). The clinical efficacy of the groups is expected to provide a basis for the clinical development of the NOSES I-type E method in combination with 3D laparoscopy to implement a sphincter preservation method for low rectal cancer with a higher probability of therapy success.

## Materials and methods

### General information

A retrospective analysis of 109 patients with laparoscopic low rectal cancer sphincter - preserving surgery without preventive stoma admitted to the Department of Colorectal Surgery in Shanxi Provincial Cancer Hospital between January 2017 and May 2019. Collected patient data included general as well as perioperative data, postoperative complications, postoperative pathological results, postoperative pain score, postoperative follow-up anal function, recurrence, and survival. According to the operation method, they were divided into the following groups: NOSES I-type E group and Dixon group according to the operation method, NOSES I-type E group further divided into 3D laparoscopy (group A) and 2D laparoscopy (group B).

Inclusion criteria: (1) Single low rectal cancer (≤7 cm distance from the anal verge) was diagnosed by preoperative digital rectal examination, colonoscopy, pathology, etc (8), and pathologically confirmed as rectal cancer; (2) Displaying stage T1-T3, judged by MR or CT with no distant metastasis; (3) All those who needed neoadjuvant therapy received neoadjuvant therapy; (4) No history of rectal anal canal disease and the tumor was not specific to the distal rectum enteritis or radiation enteritis; (5) Function well before surgery; (6) No prophylactic leakage was performed.

Exclusion criteria: (1) Preoperative or intraoperative evaluation of the tumor with a distance of greater than 7 cm from the anal verge; (2) Abnormal function of the internal and external anal sphincter before operation was excluded; (3) Malignant diseases of other systems; severe cerebrovascular disease, severe cardiopulmonary, liver and kidney dysfunction, coagulation dysfunction; (4) Abdominal and pelvic implants or distant metastases were found before or during surgery; (5) Incomplete clinical data; (6) Unable to cooperate with treatment procedures.

### Observation indicators

(1) General information: gender, age, BMI, distance from tumor to anal verge, tumor stage; (2) Perioperative indicators: operation time, intraoperative blood loss, hospitalization costs, exhaust time, eating time, catheterization time, hospital stay time, drainage tube removal time, days of analgesia; (3) Postoperative complications: anastomotic leakage, anal stenosis, rectal prolapse, colon retraction, dysuria, anastomotic bleeding, incision infection, pelvic infection, ureteral injury, incisional hernia. (4) The Visual Analogue Scale (VAS) scale was used to evaluate the degree of pain 3 days after the operation, with 0 points indicating no pain, 10 points indicating severe pain (9); (5) Postoperative pathological examination: general score type, histological type, differentiation type, T stage, N stage, specimen length, total lymph node number, distance between the lower edge of the tumor and the distal resection edge, the number of positive cases at the circumferential resection edge, the long diameter of the tumor, the width and thickness of the tumor; (6) Wexner score to evaluate anal function at 12 months after operation, which includes 5 items, each item is evaluated with 0–4 points, the total score is 0–20 points and the higher the score, the worse the anal function. 0 represents normal, Scores below 10 indicate good bowel control, 10 and above indicate incontinence, and 20 indicates complete incontinence (10); (7) 3-year local recurrence rate and distant metastasis rate; (8) 3-year survival rate. This study was approved by the Medical Ethics Committee of Shanxi Provincial Cancer Hospital (approval number: 202108), exempting informed consent.

### Surgical method

**Figure 1:**
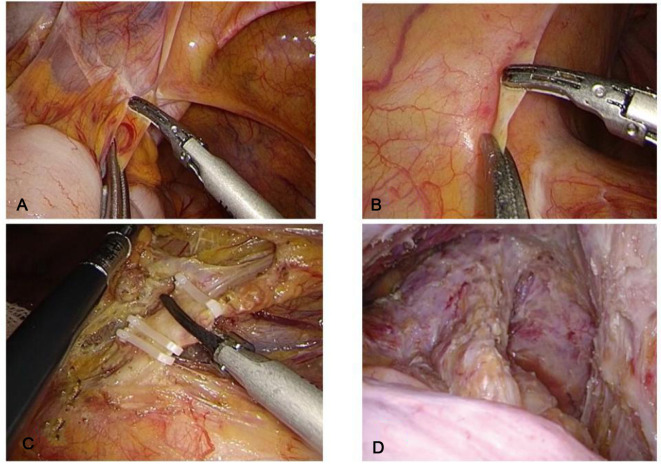
3D or 2D laparoscopy to complete the abdominal cavity operation, (**A**) Separation of the lateral physiological adhesions; (**B**) The peritoneum was incised at the level of the sacral promontory; (**C**) Disconnection of the inferior mesenteric vessels; (**D**) Dissociation to the level of the upper edge of the levator ani.

The operation followed the standard of complete total rectal mesentery resection and third-station lymph node dissection (11). The operation process mainly included: establishment of pneumoperitoneum (14 mmHg) (1 mmHg = 0.133 kPa) by the five-hole method, for which the incision was made on the front of the sacral promontory. The peritoneum, after dissecting the root of the inferior mesenteric artery, was clipped and cut off at a distance of 1 cm from the root and the inferior mesenteric vein was treated in the same way. From the back of the rectum to the plane of the coccyx tip, then free the sides and front to the level of the upper edge of the levator ani muscle. After completion of the above operations, specimen removal and bowel reconstruction were performed (Figures 1A–D).

**Figure 2:**
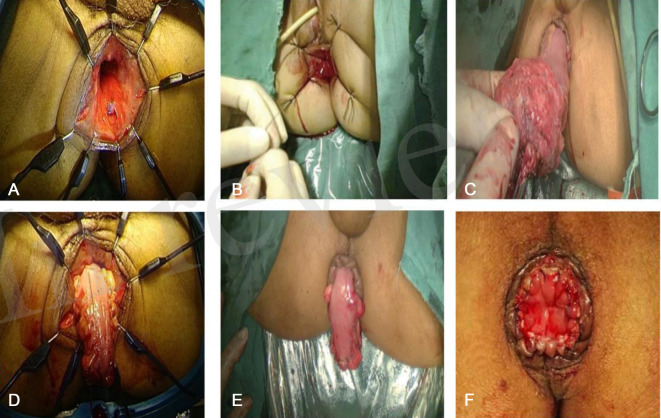
3D or 2D laparoscopic NOSES I-type E method to preserve the anus, (**A**) Full exposition of the anus; (**B**) Suturing the purse at the lower edge of the tumor; (**C**) Pulling out the rectum through the anus; (**D**) Removal of the specimen; (**E**) 5 cm of bowel was left outside the anus and fixed with suture; (**F**) Picture of 14 days after anus formation.

For the 3D or 2D laparoscopic NOSES I-type E method group the procedure was as follows: the anus was abducted with sutures, a purse-string suture 1 cm below the tumor and the anal canal mucosa was stripped above the white line or the intestinal wall was opened near the dentate line and freed upwards, retaining the internal anal sphincter and dragging the free intestinal segment down from the anus. The bowel was cut at 7–10 cm above the tumor, and 3–5 cm of bowel was left outside the anus. The surrounding sutures were fixed with 3–4 stitches, the pelvic cavity was flushed, and a drainage tube was indwelled. Stage II anoplasty was performed 14 days later (Figures 2A–F).

**Figure 3:**
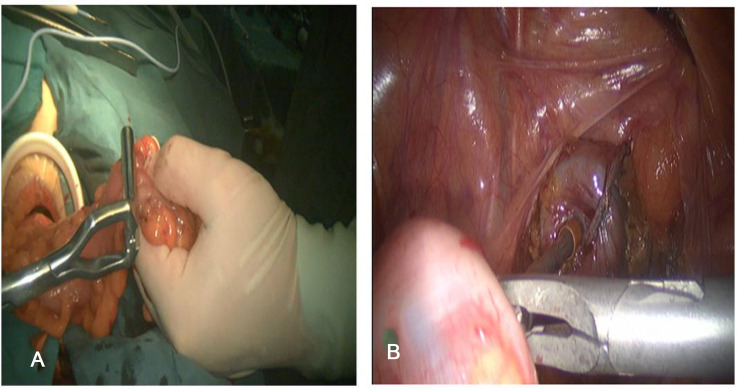
3D or 2D laparoscopic assisted Dixon method to preserve the anus, (**A**) The assisted abdominal wall incision to remove the specimen; (**B**) The colorectal anastomosis is completed through the anus.

For the 3D or 2D laparoscopic traditional Dixon group the surgery procedure was as follows: the bowel is closed with a cutting and closure device 2 cm from the lower end of the tumor, a longitudinal incision of about 5 cm is made 5 cm below the umbilicus, an incision dilator is inserted, and the proximal rectum is taken out with oval forceps, the bowel canal was transected 7 cm from the upper boundary of the tumor, the stapler base was placed, ligated in the knot groove and returned to the pelvis and the incision was closed layer by layer. The abdominal cavity was flushed with 1500 ml of normal saline under laparoscopy and no bleeding was detected (Figures 3A,B).

### Statistical methods

The data were processed by using SPSS 22.0. The count data were described by frequency and percentage and the comparison between groups was evaluated by a X^2^ or a Fisher's exact probability test; measurement data were described by mean ± standard deviation (*x ± s*). A t-test or one-way analysis of variance test was used for data analysis between groups; Mann-Whitney U test was used for nonparametric data; *P *< 0.05 indicated that the difference was statistically significantly different.

## Result

### General data comparison

A total of 109 patients successfully completed the operation with no conversion to laparotomy, no perioperative death, and postoperative pathological specimens were R0 resection. There was no significant difference in the age, gender, BMI, tumor diameter, tumor stage and other general information of the NOSES 1-type E group and Dixon group patients (*P *> 0.05), these index also have no significant difference between group A and group B (*P *> 0.05). The distance between the tumor and the anal verge was significantly different between NOSES 1-type E group group compared to Dixon group (*P *< 0.05), while there was no significant difference between group A and B (*P *>* *0.05) **(**Table 1).

**Table 1:** Comparison of the general data of the four groups.

	NOSES 1-type E group (52 cases)		Dixon group (57 cases)
	Group A (25)	Group B (27)	
Gender			
Male/Female	14/11	17/10	30/27
Age	59.61 ± 10.65	58.63 ± 8.47	60.98 ± 8.95
BMI	23.32 ± 3.12	22.28 ± 3.45	24.23 ± 2.97
Distance from anal verge	4.12 ± 0.82^a^^b^	3.98 ± 0.85^a^	5.84 ± 1.07
Tumor stage			
I	6	8	14
II	10	8	27
III	9	11	16

^a^ *P* < 0.05 Compared with Dixon group.

^b^ *P* > 0.05 Compared with group B.

### Comparison of perioperative indicators

There was no significant difference in perioperative indicators, intraoperative bleeding and operation time between NOSES I-type E group and Dixon group (*P *>* *0.05), these index also have no significant difference between group Aand group B (*P *>* *0.05). The exhaust time, eating time, drainage tube removal time, hospitalization costs, hospitalization time, the number of analgesic treatment days between NOSES I-type E group were significantly different from Dixon group (*P *< 0.05), while group A had no significant difference to group B (*P *> 0.05). There was no significant difference in catheterization time between NOSES I-type E group and Dixon group, while group A significantly less than group A (*P* < 0.05) (Table 2).

**Table 2:** Comparison of perioperative indicators.

	NOSES I-type E group (52 cases)		Dixon group (57 cases)
	Group A (25)	Group B (27)	
Intraoperative situation			
Operation time/min	188.28 ± 55.20	203.61 ± 65.18	190.87 ± 57.17
Blood loss/ml	55.11 ± 52.34	75.93 ± 63.98	67.09 ± 51.03
Postoperative situation			
Hospitalization expenses/10,000 yuan	6.45 ± 2.22^a^^b^	6.38 ± 2.21^a^	7.81 ± 2.41
Exhaust time/day	2.3 ± 0.45^a^^b^	2.70 ± 0.75^a^	2.98 ± 0.93
Meal time/day	4.0 (4.0,6.0)^a^^b^	4.0 (4.0,6.0)^a^	6.0 (4.0,8.3)
Catheterization time/day	4.0 (3.0,5.0)^c^^d^	5.0 (3.0,5.0)^c^	5.0 (3.0,9.0)
Hospital stay/day	13.30 ± 8.12^a^^b^	13.33 ± 8.17^a^	15.16 ± 10.06
Analgesia days/day	4.44 ± 1.44^a^^b^	4.52 ± 1.69^a^	4.98 ± 1.49
Drainage tube removal time/day	6.40 ± 3.81^a^^b^	6.7 ± 4.12^a^	11.41 ± 1.81

^a^ *P* < 0.05, Compared with Dixon group.

^b^ *P* > 0.05, Group A compared with group B.

^c^ *P* > 0.05, Compared with Dixon group.

^d^ *P* < 0.05, Group A compared with group B.

### Comparison of complication indicators

We compared the postoperative complications among the groups, the anastomotic leakage in NOSES I-type E group were significantly lower than Dixon group (*P* < 0.05), while there was no significant difference between groups A and B (*P* > 0.05). There were no significant difference in difficulty urinating between NOSES I-type E group and Dixon group (*P* > 0.05),while it in group A significant lower than group B (*P *< 0.05). Anal stenosis, rectal Prolapse and colonic retraction in NOSES I-type E group were significantly higher than those in Dixon group (*P* < 0.05), while there was no significant difference between group A and group B (*P* > 0.05). The occurrences of anastomotic bleedings in Dixon group higher than that of in NOSES I-type E group (*P* < 0.05), while there was no significant difference between group A and B (*P* > 0.05). There were no significant differences in the incision infection rate, numbers of pelvic infection, ureteral injury, incisional hernia, and the total complication rate among the groups (*P* > 0.05) (Table 3).

**Table 3:** Comparison of postoperative complication indicators

	NOSES I-type E group (52 cases)		Dixon group (57 cases)
	Group A (25)	Group B (27)	
Anastomotic leakage	0^a^^b^	0^a^	4/57
Anal stenosis	1 (1/25)^a^^b^	2 (2/27)^a^	0
Rectal Prolapse	0^a^^b^	1 (1/27)^a^	0
Colon retraction	1 (1/25)^a^^b^	1 (1/25)^a^	0
Difficulty urinating	0^c^^d^	1(1/27)^c^	1/57
Anastomotic bleeding	0^a^^b^	0^a^	1/30
Incision infection	0^c^^b^	0^c^	0
Pelvic infection	1(1/25)^c^^b^	1 (1/27)^c^	2/57
Ureteral injury	0^c^^b^	0^c^	0
Incisional Hernia	0^c^^b^	0^c^	0
Total	3 (3/25)^c^^b^	6 (6/27)^c^	8/57

^a^ *P* < 0.05, Comparison of NOSES I-type E group with Dixon group.

^b^ *P* > 0.05, Comparison of group A with group B.

^c^ *P* > 0.05, Comparison of NOSES I-type E group with Dixon group.

^d^ *P* < 0.05, Comparison of group A with group B.

### Postoperative pain scores

The four patient groups were scored by VAS in the first three days after operation. The results showed that NOSES I-type E group significantly lower score than Dixon group (*P* < 0.05), There was no significant difference between group A and B (*P* > 0.05) (Table 4).

**Table 4:** Comparison of pain scores in the four groups three days after operation.

	NOSES I-type E group (52 cases)		Dixon group (57 cases)
	Group A (25)	Group B (27)	
1 day	5.22 ± 0.7^a^^b^	5.14 ± 0.64^a^	6.37 ± 0.56
2 day	4.80 ± 0.68^a^^b^	4.77 ± 0.88^a^	6.05 ± 0.55
3 day	3.55 ± 0.61^a^^b^	3.58 ± 071^a^	5.41 ± 0.61

^a^ *P* < 0.05, Compared with Dixon group.

^b^ *P* > 0.05, Group A compared with and group B.

### Postoperative pathological results comparison

The postoperative pathological results of the groups showed that there were no significant differences in the gross tumor type, histological type, degree of differentiation, postoperative T stage, N stage, specimen length, total number of lymph nodes, number of cases with positive resection margin, distance from the lower tumor margin, tumor length, tumor width, and thickness between NOSES I-type E group and Dixon group (*P* > 0.05),these also have no significant differences between group A and group B (*P* > 0.05) (Table 5).

**Table 5:** Comparison of postoperative pathological indexes among the four groups.

	NOSES I-type E group (52 cases)		Dixon group (57 cases)
	Group A (25)	Group B (27)	
Gross typing			
Ulcerative	12	14	33
Invasive	7	8	14
Raised	6	5	10
Histological typing			
Adenocarcinoma	18	20	34
Adenocarcinoma/partial mucinous adenocarcinoma	3	5	12
Mucinous adenocarcinoma	4	2	11
Differentiation			
Mid-differentiation	13	16	31
Low differentiation	5	6	16
Mid-low differentiation	7	5	10
pT stage			
T1	5	4	14
T2	6	6	16
T3	14	17	17
*N* stage			
N0	16	12	24
N1	5	8	15
N2	4	7	18
Specimen length (cm)	10.20 ± 1.81	9.80 ± 2.21	10.52 ± 3.41
Total lymph nodes	13.0 ± 5.01	12.70 ± 4.56	13.11 ± 4.46
Number of positive margins	0	0	0
Distance from tumor to inferior margin	1.21 ± 0.77	1.23 ± 0.67	1.7 ± 0.80
Tumor long diameter	3.00 (1.5, 4.00)	3.00 (3.50, 4.00)	4.00 (2.75, 5.00)
Tumor width and diameter	3.00 (1.8,4.00)	3.00 (2.00,4.00)	3.00 (2.50,3.75)
Tumor thickness and diameter	1.5 (1.12,2.00)	1.5 (1.00,2.00)	1.5 (0.8,2.00)

There were no significant differences in all indexes among the groups, *P* > 0.05.

### Comparison of the anal function at 1 year after follow-up

At 1-year follow-up, the WIS score evaluated the anal function of the patients and the proportion of scores <10 points. The results showed that there was no difference in anal function among the the groups at 1 year after surgery (*P* > 0.05) (Table 6).

**Table 6:** Anal function score and the number and percentage of cases with scores less than 10 in the four groups at 1 year after operation.

	NOSES I-type E group (52 cases)		Dixon group (57 cases)
	Group A (25)	Group B (27)	
Wexner score (X ± s)	8.55 ± 1.98^a^^b^	8.21 ± 1.92^a^	7.64 ± 2.21
Wexner score <10 number and percentages	20 (20/25)^a^^b^	21 (21/27)^a^	53/57

^a^ *P* > 0.05 Compared with Dixon group.

^b^ *P* > 0.05, Comparison between group A and B.

### Comparison of recurrence and distant metastasis at 3-year follow-up

The recurrence and distant metastasis of the groups were followed up for 3 years. The results showed that in the NOSES I-type E group, there was 2 local recurrence and 2 distant metastasis whereas in Dixon group there was 3 case of distant metastasis and 1 case of local recurrence. Statistical analysis showed that there was no significant difference in recurrence and distant metastasis numbers among the groups (*P* > 0.05) (Table 7).

**Table 7:** local recurrence and metastasis in the four groups at 3 years follow-up.

	NOSES I-type E group (52 cases)		Dixon group (57 cases)
	Group A (25)	Group B (27)	
Local recurrence	1	1	1
Distant metastases	0	2	3

There was no significant difference among the groups, *P* > 0.05.

### Comparison of the survival rates at 3-year follow-up

The results showed that in the NOSES I-type E method group, 25 patients in group A and 24 patients in group B survived whereas in Dixon group, 55 patients survived. There was no significant difference in the survival rate among the groups (*P* > 0.05) (Table 8).

**Table 8:** The survival rate of the four groups at the 3-year follow-up.

	NOSES I-type E group (52 cases)		Dixon method (57 cases)
	Group A (25)	Group B (27)	
3 years alive Number	25	24	55
OS	25/25	24/27	55/57

There was no significant difference between the groups, *P* > 0.05.

## Discussion

The NOSES I-type E method is a combination of a laparoscopy and modified Bacon technique which has been described in detail earlier (12). As one of the sphincter-preserving surgical methods for low rectal cancer, it has greatly improved the sphincter-preserving rate, in addition to obtained cosmetic needs and reducing the occurrence of abdominal wall complications (13). However, the safety of the operation, the patient's anal function and prognosis are the fundamental reasons why it is difficult to reach a consensus and unification in clinical practice (14). The results of this study showed that there was a significant difference in the distance between the tumor and the anal verge in the general data of the NOSES I-type E group and the Dixon group. It shows that the distance between the tumor and the anal verge is related to the surgical method (15) and the NOSES I-type E method may be more suitable for anus preservation in ultra-low rectal cancer. This result is also in line with the current domestic expert consensus.

The perioperative indicators showed that compared to the Dixon method, the NOSES I-type E method patients had a better feeding time, exhaust time, drainage tube removal time, hospitalization time, hospitalization costs, and less postoperative analgesia days. Moreover, NOSES I-type E method patients displayed significantly lower postoperative complications. The pain score of patients in the first three days after operation was significantly lower in the NOSES I-type E method patients compared to the Dixon method patients. These results showed that the NOSES I-type E method had no anastomotic stoma, so there is no need to worry about anastomotic leakage and the patient can be instructed to eat and get out of bed early, and the recovery of gastrointestinal function was promoted. Another major advantage of the NOSES I-type E method is the fact that there was no assisted incision in the abdominal wall, therefore, the pain level of the patients was significantly lower than that of the Dixon method. This is why the patients were more motivated to get out of bed actively, so as to promote the rapid recovery which eventually resulted in a significant reduction in the length of hospital stay and postoperative analgesia days. The idea is consistent with NOSES (16). In addition, NOSES I-type E surgery does not need a stapler and closure device. The hospitalization time is shortened and the hospitalization costs are significantly lower than that of the Dixon method.

Our results also indicate that the difficulty urinating in NOSES I-type E group has no significant difference compared to Dixon group, but it has significant difference between group A and group B. which reveled that due to the advantages of 3D laparoscopy, there was a better pelvic nerve protection. Thus, the NOSES I-type E method can achieve the same neurological protection as Dixon surgery (17). The surgical complications showed that the NOSES I-type E method group were higher than those in the traditional Dixon group, which indicated that the NOSES I-type E method also had its shortcomings, however, there was no statistical difference in the total complication rate among groups. This is consistent with already published data (18).

There was no significant difference in postoperative pathological results between the groups. The two surgery techniques can achieve the same radical efficiency under the guidance of TME and D3 surgery principles. However, poor anal function and difficulty in achieving satisfactory stool control after NOSES I-type E method have always been concerns of surgeons. The results of this study showed that there was no significant difference in anal function scores between the two method one year after operation. This is consistent with the research results of Liu, Li (19), and Luo Xue (20), and others which reported that the patient's anal function after NOSES I-type E surgery achieved the same results as the Dixon operation technique 3 and 6 months after operation (21). This indicated that after 1 year of muscle and nerve function recovery, the two groups of patients can achieve the same therapeutic effect. We should not focus on short-term anal function but should observe a certain time limit. The results of this study also showed that the recurrence and overall survival rate 3 years after surgery were not statistically different between the two groups, which was consistent with the results of previous studies (22, 23). These data show that the two surgical methods can achieve the same therapeutic effect with comparable safety and surgical efficiency.

As on of the largest colorectal cancer diagnosis and treatment center in China, it is currently the center that has carried out more NOSES I-type E method in China. The experience of this center is as follows: (1) Select suitable patients according to tumor characteristics such as stage c/ycT1–3, distance of the tumor 3–5 cm away from the anal verge, involving no more than half of the intestinal wall with a tumor diameter less than 3 cm, early cancer or carcinoma *in situ* where local anal resection cannot be performed; male patients with preoperative perianal muscles are selected for NOSES I-type E method (2) During abdominal surgery, the sigmoid colon needs to obtain sufficient mobility, must be released upward to the splenic flexure of the colon and the rectum must be freed downward to the levator ani muscle or between the internal and external sphincter; (3) The anus should be fully expanded, the rectal anal canal should be disinfected and the purse-string suture at the distal end of the tumor should be free of tumors; (4) The skin of the anal canal should be incised 1 cm below the dentate line and all the mucosa and abdominal cavity should be removed upwards; (5) After specimen removal, the proximal colon should be pulled out through the anus for about 5 cm and the intestinal seromuscular layer and the skin of the anal canal should be sutured intermittently, which requires major intestinal tension and blood supply to prevent postoperative ischemia, necrosis, and retraction; (6) Anus reconstruction should be performed 10 days after the first operation. The seromuscular layer should be incised close to the anal margin and the mucosa should be 0.5 cm longer than the anal margin; (7) Postoperative levator training should be performed to restore muscle function around the anus.

In conclusion, the NOSES I-type E method can achieve the same radical and prognostic effect as the Dixon operation without increasing surgical complications while at the same time has the advantages of no anastomotic leakage, avoidance of permanent abdominal stoma, less trauma, and complete preservation of anal function. Its combination with 3D laparoscopy can better preserve the patient's neurological function than 2D laparoscopy. Of course, the NOSES I-type E method also has certain shortcomings, such as postoperative anal stenosis, colon retraction, and short-term poor anal function. But no surgery is perfect, only the right one is the best. Therefore, surgeons should accurately and individually assess the patient's condition and, based on their own experience, choose the NOSES I-type E method only for suitable patients. This study also has certain limitations, such as the small number of cases, the short follow-up time, and the specific survival curves of the groups. These deficiencies will be further investigated in future studies.

## Conclusions

The NOSES I-type E method is a safe and effective sphincter-preserving operation for low rectal cancer and its combination with 3D laparoscopy may have better neurological protection which is worth of clinical application.

## Data Availability

The original contributions presented in the study are included in the article/Supplementary materials, further inquiries can be directed to the corresponding author/s.
